# Schoolteachers with voice handicap are twice as likely to report depressive symptoms

**DOI:** 10.1007/s00405-022-07376-w

**Published:** 2022-04-20

**Authors:** Alberto Durán González, Ana Carolina Bertin de Almeida Lopes, Selma Maffei de Andrade, Flávia Lopes Gabani, Mayara Cristina da Silva Santos, Renne Rodrigues, Arthur Eumann Mesas

**Affiliations:** 1 Postgraduate Program in Public Health, Health Sciences Center, State University of Londrina, Av. Robert Koch, n° 60, Londrina, 86038-350 Brazil; 2 Department of Nursing, Health Sciences Center, State University of Londrina, Londrina, Brazil; 3 Universidad de Castilla-La Mancha, Health and Social Research Centre, Cuenca, Spain

**Keywords:** Depression, Depressive symptoms, Voice disorders, Teachers, Epidemiology

## Abstract

This study aimed to examine the association between voice disorder and depressive symptoms in schoolteachers. A cross-sectional survey was conducted with elementary and secondary schoolteachers. Voice disorders and depressive symptoms were assessed with the Voice Handicap Index-10 and the Beck Depression Inventory-II, respectively. Logistic and linear regressions models were adjusted for confounding variables. In the 389 schoolteachers studied, the prevalence of voice handicap and depressive symptoms was 18.8 and 38.8%, respectively. Voice handicap was associated with depressive symptoms on all models tested. The logistic regression showed an odds ratio of 2.21 (95% confidence interval: 1.19, 4.08; *p* value < 0.05), while in the linear regression each point increase on the voice disorder scale increased the Beck Depression Inventory-II score by 0.39 points (95% confidence interval: 0.26, 0.54; *p* value < 0.05). This study showed that teachers of public schools with voice handicap are twice as likely to report depressive symptoms.

## Introduction

Voice disorders are very common in teachers, who usually have a high demand on their voice at work [1, 2]. It has been estimated that the prevalence of voice disorders in teachers is two- to three-fold higher than the prevalence in the general population [1]. Risk factors for voice disorders in teachers include environmental conditions (acoustics, noise, humidity, dust and temperature), organizational characteristics (long work hours, excessive number of students per classroom, multiple jobs and excessive voice demand), biological factors (allergies, reflux and infections of the upper respiratory tract), and psychological disorders (depression and anxiety) [1–6].

Mental health problems, such as depression, are highly prevalent worldwide and may have serious health and socioeconomic implications. The etiology of depression is multifactorial and has not been completely elucidated. Genetic, biological, psychological and psychosocial conditions are factors that have been implicated in the development of depression, in addition to work-related conditions and lifestyle risk factors [7–9]. Epidemiological studies have pointed out that teachers are more likely to develop psychological disorders such as stress, anxiety, depression and irritability because of work-related stressful conditions [10, 11].

Some reports highlight the high prevalence of anxiety, stress and depressive symptoms in individuals presenting voice disorders [11, 12]. In general, these studies support that there is a link between voice and psychological disorders [13–15]. Cantor Cutiva and Burdof [16] reported that the severity of voice complaints, as well as auditory symptoms and hearing impairment, are associated to teachers' perception of quality of life [16]. Vanhoudt et al. stated that teachers with a high voice handicap were at a greater risk for depression compared with those with lower voice handicap [17]. Those authors suggested that a depressive attitude could arise when teachers recognize that voice disorders influence their performance in the classroom [17]. A longitudinal survey with female student teachers revealed that having a higher voice handicap predisposed them to have higher anxiety scores after four years of follow-up [18].

In this context, voice disorders have arisen as a factor potentially involved in the development of mental health problems in voice professionals [14]. The elucidation of such a relationship is particularly important in schoolteachers since these professionals are at a greater risk of developing voice problems [1, 19], which may impact their professional performance and lead to social, psychological, physical and economic consequences [19]. We found four studies on this subject in teachers, and all supported the relationship between voice and mental disorders [17, 20–22]. All studies used questionnaires designed to assess composite mental outcomes such as “common mental disorder symptoms” [20], biopsychosocial dimensions including depression [17], the mental summary score of health-related quality of life [21], and major depression episode, general anxiety disorder, phobias and post-traumatic stress disorder [22]. Thus, the association between voice disorder and depressive symptoms, assessed with reliable instruments specifically validated to detect these symptoms, is still lacking.

Therefore, we aimed to investigate whether voice disorders are associated with depressive symptoms in schoolteachers. Based on the available evidence, we hypothesized that this association will be confirmed, as those with voice disorders would be more prone to suffer from psychological disorders, such as depressive symptoms. In addition, we verified to what extent the main study association varies according to the sociodemographic, lifestyle and health conditions.

## Methods

### Participants and procedures

A cross-sectional study, nested in a fixed cohort, was carried out with schoolteachers from elementary and secondary levels of education of 20 public schools with the largest number of teachers (i.e., those with more than 70 teachers), located in the urban area of Londrina, a city in southern Brazil, with approximately 570 000 inhabitants (www.ibge.gov.br). These schools were selected because they are workplaces for approximately 70% (*n* = 1.126) of the total teachers at public schools in the city and because they are located in all city regions, allowing inferences that the results would apply to the entire city. Data were collected through scheduled interviews from August 2014 to March 2015 as part of the second phase of a research project entitled PRO-MESTRE–Health, Lifestyle and Work of Schoolteachers of the Public System of Parana State, which aimed to evaluate health, lifestyle and working aspects of public schoolteachers [5]. The Human Research Ethics Committee of the State University of Londrina approved this study, and all the participants were informed about the goals of the study and provided written informed consent before data collection.

### Inclusion and exclusion criteria

In the first phase of the project, 978 (response rate of 92.2%) teachers were interviewed. The inclusion criteria for the second phase were the teachers participating in the first phase who worked in the classroom and who were in charge of a subject in class. In the second phase 488 individuals failed to attend the follow-up interviews due to a major labor strike and were missed. Among the 490 teachers eligible for the study, there were 60 losses due to 20 refusals, 16 losses after three attempts to interview, 14 moved out of the city, nine were not found, and one had been arrested, resulting in 430 teachers interviewed. We further excluded 41 teachers who lacked information on the Voice Handicap Index-10 (VHI-10) and/or Beck Inventory. Thus, the final sample of our study was 389 (39.8% of phase one) schoolteachers.

### Voice disorder

Voice disorder was assessed with the Voice Handicap Index 10 (VHI-10) [23], a validated vocal function assessment tool that consists of 10 items in which the participant rates each item using a Likert scale (0–never; 1–almost never; 2–sometimes; 3–almost always; 4–always). The sum of the answers to each item resulted in a total score reflecting the handicap related to voice problems. According to the literature, we defined a cut-off point of VHI-10 score above 11 to consider an abnormal result [24]. Accordingly, we considered schoolteachers with scores greater than 11 as having voice disorders and those with total scores equal to or less than 11 as not having voice disorders.

### Depressive symptoms

Depressive symptoms were evaluated with the Beck Depression Inventory II (BDI-II) [25], a self-reported questionnaire with 21 items about depressive symptoms in the last 15 days. The BDI-II was validated for application in a Brazilian non-clinical population and is considered a valid and reliable tool for this purpose [26]. Each item is composed of four alternatives (0, 1, 2 or 3), revealing increasing levels of depressive symptoms. The sum of the scores varies from 0 to 63 points, resulting in four categories: absence of or minimal depressive symptoms (0–13), mild depressive symptoms (14–19), moderate depressive symptoms (20–28), and severe depressive symptoms (29 and above). In the present study, the variable was dichotomized for analysis in the presence of depression symptoms (scores above 13) or absence or minimal depressive symptoms (score 13 or lower).

### Sociodemographic variables

Sociodemographic characteristics were collected from a self-administered questionnaire and included sex, age (categorized as ≤ 40 or > 40 years old), marital status (living with or without a partner), and monthly family income in Brazilian currency (up to USD 1.119,00, USD 1.120,00 to 2.611,00 and higher than R$ 2.611,00) (1 USD = 2.68 Brazilian Real, at the middle point of data collection, January 2015).

### Lifestyle and health conditions variables

Lifestyle variables included in the analysis were smoking status (non-smoker/former smoker or current smoker) and alcohol intake (drink or do not drink alcohol beverages). Health conditions considered in the study were categorized as follows: sleep quality (very good/good or bad/very bad), chronic pain—pain lasting for more than 6 months (yes or no), anxiety—medical diagnosis (yes or no), and self-rated health (very good/good or regular/bad/very bad).

### Work-related variables

Information on teaching characteristics and work conditions was obtained through interviews and comprised the teacher’s educational level (bachelor’s degree, specialization or master’s degree/PhD), years of teaching (≤ 5, 6 to 10, 11 to 15, or > 15 years), teaching levels (elementary, elementary and other levels), perception of the number of students per classroom (excellent/good or bad/very bad), hours of teaching per week (< 40 or ≥ 40 h), and perception of noise affecting or not affecting their work (noise within the classroom, in the courtyard, hallways and sports court, and out-of-school).

### Statistical analysis

The chi-square test was used to examine associations between depressive symptoms and sociodemographic, lifestyle, health conditions, and work-related variables. Multiple logistic regression models were performed to test whether voice disorder (main independent variable) was associated with depressive symptoms (dependent variable). Variables with a *p* value < 0.20 in the bivariate analysis were included in the regression models, except age and alcohol intake, which was included based on the literature results, showing that age may contribute to the prevalence of depressive symptoms, especially in women [27, 28], and alcohol intake has been associated with the risk of depression in cohort studies[29]. Additionally, linear regression was performed between the continuous scores of the Beck Depression Inventory-II and the Voice Handicap Index 10. For both models, multiple logistic and linear regression, we added the covariates to the models in three separate blocks: Model 1: sex and age; Model 2: model 1 + smoking status, alcohol intake, chronic pain, sleep quality, anxiety and self-rated health; Model 3: model 2 + educational level, teaching levels, perception of the number of students per classroom and noise within the classroom. We also tested for interaction between voice disorder and sex and voice disorder and age by adding interaction terms to the fully adjusted model. The results of the interaction terms did not show statistical significance, and therefore, the existence of effect modification on the main association was disregarded.

All statistical analyses were conducted in Stata Software version 13.1 (StataCorp, College Station, TX, USA), and tests with *p* values < 0.05 were considered statistically significant.

## Results

A total of 389 school teachers (65.5% women, mean age of 41.6 ± 9.4 years) were included in the analyses. The prevalence of voice disorders and depressive symptoms was 18.8 and 38.8%, respectively. Among those with depressive symptoms (*n* = 151), 66 (43.7%) were classified as having mild depressive symptoms, 52 (34.4%) as having moderate depressive symptoms, and 33 (21.9%) as having severe depressive symptoms.

In the bivariate analysis, depressive symptoms were more frequent in women than in men (*p* = 0.002), while that frequency did not vary across the categories of other sociodemographic characteristics. In addition, female teachers had a significantly higher frequency of voice disorders (22.4%) than male teachers (11.9%) (*p* = 0.012). Depressive symptoms were more frequent in those with poor sleep quality, along with those who reported chronic pain, clinical diagnosis of anxiety, and negative self-rated health (*p* < 0.05). Considering work-related characteristics, depressive symptoms were more frequent in schoolteachers who reported that noise within the classroom affected their work (*p* = 0.006) and in those with voice disorders (*p* < 0.001) (Table 1).

**Table 1:** Sociodemographic, lifestyle, health and work conditions, teaching characteristics, and voice disorders according to the presence of depressive symptoms in Brazilian schoolteachers

Variables	Total (389)		Depressive symptoms*		*p* value^†^
			Yes	No	
	*n*	%	*n* (%)	*n* (%)	
Sex					
Men	134	34.5	38 (28.4)	96 (71.6)	0.002
Women	255	65.5	113 (44.3)	142 (55.7)	
Age (years)					
≤ 40	140	36.0	57 (40.7)	83 (59.3)	0.565
> 40	249	64.0	94 (37.7)	155 (62.3)	
Marital status					
Live with a partner	239	61.4	94 (39.3)	145 (60.7)	0.793
Live without a partner	150	38.6	57 (38.0)	93 (62.0)	
Monthly family income (USD)^**‡**^					
≤ 1.119,00	39	10.5	18 (46.2)	21 (53.8)	0.313
1.120,00 to R$ 2.611,00	215	57.8	87 (40.5)	128 (59.5)	
> 2.611,00	118	31.7	40 (33.9)	78 (66.1)	
Smoking status^**‡**^					
Non-smoker/former smoker	352	91.0	132 (37.5)	220 (62.5)	0.107
Current smoker	35	9.0	18 (51.4)	17 (48.6)	
Alcohol intake^**‡**^					
No	227	59.6	91 (40.1)	136 (59.9)	0.546
Yes	154	40.4	57 (37.0)	97 (63.0)	
Sleep quality^**‡**^					
Very good/good	283	72.9	85 (30.0)	198 (70.0)	< 0.001
Bad/very bad	105	27.1	65 (61.9)	40 (38.1)	
Chronic pain^‡^					
No	205	52.8	69 (33.7)	136 (66.3)	0.025
Yes	183	47.2	82 (44.8)	101 (55.2)	
Diagnosis of anxiety					
No	309	79.4	107 (34.6)	202 (65.4)	0.001
Yes	80	20.6	44 (55.0)	36 (45.0)	
Self-rated health^**‡**^					
Very good/good	303	78.9	97 (32.0)	206 (68.0)	< 0.001
Bad/very bad	81	21.1	53 (65.4)	28 (34.6)	
Educational level^**‡**^					
Diploma	37	9.6	9 (24.3)	28 (75.7)	0.050
Specialization	295	76.2	124 (42.0)	171 (58.0)	
Master/PhD	55	14.2	17 (30.9)	38 (69.1)	
Years of teaching					
≤ 5	65	16.7	30 (46.2)	35 (53.8)	0.543
6–10	78	20.0	31 (39.7)	47 (60.3)	
11–15	122	31.4	46 (37.7)	76 (62.3)	
> 15	124	31.9	44 (35.5)	80 (64.5)	
Teaching levels					
Elementary	70	18.0	32 (45.7)	38 (54.3)	0.191
Elementary and other levels	319	82.0	119 (37.3)	200 (62.7)	
Perception of the number of students per classroom					
Excellent/good	105	27.0	34 (32.4)	71 (67.6)	0.113
Bad/very bad	284	73.0	117 (41.2)	167 (58.8)	
Weakly workload^‡^					
< 40 h	142	37.7	60 (42.2)	82 (57.8)	0.354
≥ 40 h	235	62.3	88 (37.4)	147 (62.6)	
Noise within the classroom					
Does not affect	35	9.0	6 (17.1)	29 (82.9)	0.006
Affect	354	91.0	145 (41.0)	209 (59.0)	
Noise in courtyard, hallways and sports court					
Does not affect	104	26.7	35 (33.6)	69 (66.4)	0.207
Affect	285	73.3	116 (40.7)	169 (59.3)	
Out-of-school noise					
Does not affect	200	51.4	74 (37.0)	126 (63.0)	0.449
Affect	189	48.6	77 (40.7)	112 (59.3)	
Voice disorder (VHI–score > 11)					
No	316	81.2	107 (33.9)	209 (66.1)	< 0.001
Yes	73	18.8	44 (60.3)	29 (39.7)	

*Depressive symptoms were classified according to the Beck Depression Inventory-II: score 0–13, no for depressive symptoms; score ≥ 14, yes for depressive symptoms

^†^Chi-square test

^‡^Data missing for some participants

Table 2 summarizes the logistic regression models performed to test the association between voice disorders and depressive symptoms. Voice disorder was significantly associated with depressive symptoms in all analyses. The adjusted odds ratio for depressive symptoms was 2.21 (95% confidence interval: 1.19, 4.08) for teachers with voice disorders compared to those without this condition, as observed in the fully adjusted model. Each point increase on the voice disorder scale increased the Beck Depression Inventory-II score by 0.39 points (95% CI 0.26, 0.54) (Table 3). Other covariates significantly associated with depressive symptoms in the complete adjusted model were women, poor sleep quality, bad/very bad self-rated health and perception that noise within the classroom affected work.

**Table 2:** Logistic regression models to estimate the odds ratio (OR) and 95% confidence interval (CI) of depressive symptoms (dependent variable) according to voice disorders (main independent variable) in Brazilian schoolteachers

Variable	Unadjusted	Model 1^a^	Model 2^b^	Model 3^c^
Voice disorder (VHI–score > 11)				
No	1.00	1.00	1.00	1.00
Yes	2.96 (1.75, 5.00)*	2.90 (1.69, 4.96)*	2.23 (1.23, 4.03)*	2.21 (1.19, 4.08)*
Sex				
Men		1.00	1.00	1.00
Women		1.94 (1.22, 3.10)*	1.88 (1.11, 3.21)*	1.74 (1.01, 3.01)*
Age (years)				
≤ 40		1.00	1.00	1.00
> 40		0.70 (0.45, 1.09)	0.66 (0.39, 1.09)	0.65 (0.38, 1.08)
Smoking status				
No smoker/former smoker			1.00	1.00
Current smoker			1.26 (0.55, 2.88)	1.63 (0.68, 3.91)
Alcohol intake				
No			1.00	1.00
Yes			1.11 (0.67, 1.84)	1.11 (0.66, 1.85)
Sleep quality				
Very good/good			1.00	1.00
Bad/very bad			3.40 (1.99, 5.80)*	3.32 (1.92, 5.72)*
Chronic pain				
No			1.00	1.00
Yes			1.14 (0.69, 1.87)	1.14 (0.69, 1.89)
Diagnosis of anxiety				
Yes			1.00	1.00
No			1.59 (0.88, 2.85)	1.41 (0.77, 2.57)
Self-rated health				
Very good/good			1.00	1.00
Bad/very bad			2.82 (1.56, 5.09)*	2.89 (1.58, 5.29)*
Educational level				
Diploma				1.00
Specialization				1.89 (0.78, 4.63)
Master/PhD				1.21 (0.42, 3.51)
Teaching levels				
Elementary				1.00
Elementary and other levels				0.79 (0.42, 1.48)
Perception of the number of students per classroom				
Good/excellent				1.00
Bad/very bad				1.19 (0.70, 2.04)
Perception of noise within the classroom				
Does not affect				1.00
Affect				3.12 (1.16, 8.37)*

*p value < 0.05

^a^Model 1: Adjusted for sex and age

^b^Model 2: Adjusted for Model 1 + smoking status + alcohol intake + sleep quality + chronic pain + diagnosis of anxiety and self-rated health

^c^Model 3: Adjusted for Model 2 + educational level + teaching levels + perception of the number of students in the classroom and noise within the classroom

**Table 3:** Linear regression models to estimate the Beta and 95% confidence interval (CI) of Beck Depression Inventory-II (dependent variable as a continuous variable) according to Voice Handicap Index 10 (main independent variable as a continuous variable) in Brazilian schoolteachers

Variable	Unadjusted	Model 1^a^	Model 2^b^	Model 3^c^
Voice handicap index 10	0.58 (0.43, 0.73)*	0.57 (0.42, 0.72)*	0.49 (0.26, 0.55)*	0.39 (0.26, 0.54)*

^a^Model 1: Adjusted for sex and age

^b^Model 2: Adjusted for Model 1 + smoking status + alcohol intake + sleep quality + chronic pain + diagnosis of anxiety and self-rated health

^c^Model 3: Adjusted for Model 2 + educational level + teaching levels + perception of the number of students in the classroom and noise within the classroom

## Discussion

This study with schoolteachers from elementary and secondary Brazilian schools found that voice disorder was associated with depressive symptoms after adjustment for sociodemographic, lifestyle, health and work-related covariates. The synergistic associations between voice and mental disturbances may have consequences not only for professionals but also for school management and, thus, the entire educational process, as such conditions can increase absenteeism among teachers [20–22].

These results are consistent with previous studies using the VHI and different criteria to define mental health symptoms. For instance, da Rocha and Mattos Souza found that emotional, functional, and organic voice handicap scores were significantly higher in teachers with common mental disorder symptoms as measured with the Self-Reporting Questionnaire [20]. Similar results were found by Moy et al. using the SF12-v2 mental component summary score [21] and Vanhoudt et al. with the Symptom Checklist-90 tool, which comprises several biopsychosocial dimensions, including depression [17]. Another study by Nerrière et al. used a questionnaire (Composite International Diagnostic Interview Short Form) to simultaneously detect major depression episodes, general anxiety disorder, phobias and post-traumatic stress disorder [22]. In their results, these last authors reported that those who reported voice disorders presented a higher level of psychological distress, as estimated in sex- and age-adjusted significant results for a major depressive episode, general anxiety disorder and phobia [22].

To the best of our knowledge, no longitudinal studies in active teachers have explored the relation between voice and depressive complaints using validated and specific tools. To date, Meulenbroek et al. studied female student teachers throughout their education and found a tendency for a positive relationship between higher emotional voice handicap and more psychosomatic complaints [18]. More specifically, students with higher total scores of voice handicap, along with those with higher points on the VHI-emotional subscale, had three to five times more risk of having anxiety and agoraphobia and almost four times higher risk of having depressive symptoms. The authors expressed that depression may be a consequence of intense and prolonged anxiety and that a set of dysfunctions, including vocal, social and cognitive problems, can be concurrent with depression [18]. However, the direction of the association is still unclear among these professionals, and prospective research is required to analyze whether voice disorder leads to depressive symptoms over time and whether this is a bidirectional association. Two recent longitudinal studies support this last hypothesis. First, a study carried out with teachers of municipal schools from Pelotas, Brazil, found that teachers who reported a voice disorder had an increased risk of developing common mental disorders [12]. On the other hand, the same research group reported that teachers who had both a voice disorder and symptoms of a common mental disorder were more likely to maintain the voice disorder through the follow-up [12].

Most studies with teachers have fewer male participants since this profession is more traditionally developed by women. Furthermore, depressive symptoms are more prevalent in women than in men in the general population [28] and among teachers [30]. Some intrinsic biological characteristics are identified as potential risk factors for increased rates of voice problems among women, such as constant hormonal influence; changes in glottic configuration during phonatory condition after a period of loud reading; less hyaluronic acid (a macromolecule that plays an important role in vocal fold performance) in the most superficial depth of the lamina propria, predisposing them to voice problems; and finally, women have a narrower larynx, requiring them to speak with a louder voice to be heard [31–33]. However, the present results showed a significant association between voice disorder and depressive symptoms regardless of sex. This suggests that the relationship between voice disturbances and mental health is similar in both sexes, and the understanding of the coexistence of these symptoms requires extending the knowledge further than sex-related issues.

The prevalence of voice disorders in teachers is much higher than that observed in the general population [1, 2] and increases according to the time dedicated to this profession [34, 35]. In addition to working as a teacher, classroom conditions, excessive noise, and individual health conditions, habits, and addictions are also considered risk factors for the development of dysphonia [1, 2]. Although in the present results self-rated health and the perception of noise within the classroom remained associated with depressive symptoms in the full adjusted model, the association between voice disorder and depressive symptoms remained regardless of age, health conditions and some occupational aspects. Therefore, other sociodemographic, lifestyle, health and occupational potential confounders not addressed in this study may be considered in future research. To date, this is the first study that included sleep quality because there is evidence that voice disturbances and sleep complaints are related to each other [36] and, in addition, some evidence pointed out that voice functioning is also related to sleep quality [37]. We found a substantial association between sleep quality and depression, although the association between depression and voice handicap remained regardless of adjusting for sleep quality.

Currently, it is understood that voice and psychological disorders in teachers may coexist and that when those with voice handicap fail to report voice problems, they are at higher risk for disorders such as depression and anxiety [17, 22]. Furthermore, when teachers are aware of the impact of voice disorders on their performance, they may assume a depressive attitude [17]. Although the mechanisms behind such association are still unclear, it was suggested that the coping style of teachers with a relatively high VHI score may lead to less optimal problem-solving possibilities and consequently increased vulnerability, health problems and less psychological well-being [38]. In a recent systematic review, the voice was pointed out as a potential marker of mental disorders [39], reinforcing the need for more studies in this field of knowledge.

Some methodological aspects must be considered in the interpretation of our findings. First, the cross-sectional study design limits inferences regarding the direction of the association between voice disorder and depressive symptoms. Second, although the present analysis was adjusted for several potential confounders, residual confounding is still possible, and the categorical use of some variables, such as alcohol intake and noise perception, may have reduced the adjustment of the models performed. All study information was self-reported and there was a considerable loss of participants in the second phase of the research. Finally, there was a considerable loss of participants due to a state labor strike, which could affect the representativeness of the analyzed sample. However, the VHI-10 is a widely used tool for evaluating voice disorders. Although this instrument does not provide evidence of a clinical diagnosis, it shows good efficiency in rating voice disorders, with a reduced number of items compared with the original VHI-30 items [40, 41]. In addition, the BDI-II is a reliable and valid instrument commonly used to measure depressive symptomatology in nonclinical populations [25, 26]. Certainly, differences in the specific criteria used to measure both conditions may contribute to the magnitude of the results observed among studies.

## Conclusion

In summary, this research highlights the importance of voice disorders as a potential predictor of depressive symptoms and stimulates longitudinal studies to be carried out to clarify the temporal relationship between voice complaints and depression symptoms. These findings endorse that health problems are complex and multidimensional, linking physical or somatic complaints with psychological factors, which is accentuated depending on the professional setting. Understanding the factors involved in such morbidities is necessary to reduce and prevent their occurrence. A multidisciplinary approach to implement successful treatment and preventive programs for teachers to reduce these health problems should also be emphasized.

## Data Availability

The datasets generated during and/or analyzed during the current study are available from the corresponding author on reasonable request.
